# Exercise improves cognitive dysfunction and neuroinflammation in mice through Histone H3 lactylation in microglia

**DOI:** 10.1186/s12979-023-00390-4

**Published:** 2023-11-17

**Authors:** Hao Han, Yawei Zhao, Junda Du, Sushan Wang, Xuehan Yang, Weijie Li, Jiayi Song, Siwei Zhang, Ziyi Zhang, Yongfei Tan, Grant M. Hatch, Ming Zhang, Li Chen

**Affiliations:** 1 Key Laboratory of Pathobiology, Ministry of Education, College of Basic Medical Sciences, Jilin University, Changchun, 130021 China; 2 Department of Pharmacology, College of Basic Medical Sciences, Jilin University, 126 Xin Min Street, Changchun, 130021 Jilin China; 3 School of Pharmaceutical Sciences, Jilin University, Changchun, 130021 China; 4 The Second Hospital of Jilin University, Changchun, 130041 China; 5 South China Institute of Collaborative Innovation, Dongguan, 523808 China; 6 Departments of Pharmacology and Therapeutics, Biochemistry and Medical Genetics, Center for Research and Treatment of Atherosclerosis, DREAM Children’s Hospital Research Institute of Manitoba, University of Manitoba, Winnipeg, MB R3E0T6 Canada

**Keywords:** Exercise, Lactate, Lactylation, Microglia, Neuroinflammation, Alzheimers disease

## Abstract

**Background:**

Exercise is postulated to be a promising non-pharmacological intervention for the improvement of neurodegenerative disease pathology. However, the mechanism of beneficial effects of exercise on the brain remains to be further explored. In this study, we investigated the effect of an exercise-induced metabolite, lactate, on the microglia phenotype and its association with learning and memory.

**Results:**

Microglia were hyperactivated in the brains of AlCl_3_/D-gal-treated mice, which was associated with cognitive decline. Running exercise ameliorated the hyperactivation and increased the anti-inflammatory/reparative phenotype of microglia and improved cognition. Mice were injected intraperitoneally with sodium lactate (NaLA) had similar beneficial effects as that of exercise training. Exogenous NaLA addition to cultured BV2 cells promoted their transition from a pro-inflammatory to a reparative phenotype.

**Conclusion:**

The elevated lactate acted as an “accelerator” of the endogenous “lactate timer” in microglia promoting this transition of microglia polarization balance through lactylation. These findings demonstrate that exercise-induced lactate accelerates the phenotypic transition of microglia, which plays a key role in reducing neuroinflammation and improving cognitive function.

**Supplementary Information:**

The online version contains supplementary material available at 10.1186/s12979-023-00390-4.

## Introduction

Dementia is a progressive neurodegenerative disease that causes cognitive impairment in older people. Alzheimer’s disease (AD) is one of the most common forms of dementia characterized by the extracellular deposition of amyloid β (Aβ) and intracellular aggregation of hyperphosphorylated tau, as well as microglia-mediated neuroinflammation [1–3]. Examination of the mechanisms of the microglia inflammatory response in AD and other neurodegenerative diseases may offer new intervention strategies.

Microglia play an integral role in central nervous system (CNS) tissue maintenance, injury response, and pathogen defense. Microglia serve as resident macrophage which dynamically survey the brain environment. They become activated in response to Aβ peptides, neurotoxin and proinflammatory mediators during the progression of AD [4–6]. Activated microglia exhibit abnormal morphology and proliferation and release inflammatory and bioactive molecules, which may damage neurons and exacerbate AD progression [7]. Microglia exist in different states including anti-inflammatory/reparative or pro-inflammatory, the reparative microglia express cytokines and receptors that inhibit inflammation and restore homeostasis [8, 9]. However, recent studies on microglia have shown that they are highly plastic, for example, the same microglia cell may exhibit both anti-inflammatory and pro-inflammatory markers [10, 11]. Thus, directing microglia cells towards a reparative phenotype may be a promising strategy to modulate AD progression.

Physical exercise is associated with neuronal protection and anti-aging and is recommended as a preventive and therapeutic non-drug strategy for patients with neurodegenerative diseases [12–14]. Exercise may modulate the activation state of microglia to prevent neuroinflammation in the CNS [15]. However, the mechanism remains unknown. Recent studies have suggested that exercise induces elevated levels of several cytokines, humoral factors, and metabolites that benefit the CNS in a paracrine or endocrine manner [16–22]. For example, blood from exercised mice can promote adult hippocampal neurogenesis, reduce neuroinflammation and improve cognitive performance in the sedentary mice [23, 24]. This indicates that there may be some neuroprotective exercise-induced factors in the circulation involved in the regulation of the physiological function of microglia, which is worth further elaboration.

Lactate is a major product of exercise and is transported by blood to various tissues throughout the body [25–27]. It’s transported by the lactate transporters (MCTs) across the blood–brain barrier (BBB) to multiple brain regions including the hippocampus [28, 29]. High-intensity interval exercise training induces the upregulation of vascular endothelial growth factor A (VEGFA) in the brain through lactate receptor (HCAR1) and promotes angiogenesis [30]. Voluntary exercise induces lactate accumulation in the hippocampus, where it promotes learning and memory formation by inducing brain-derived neurotrophic factor (BDNF) expression through SIRT1-dependent induction of the PGC1a/FNDC5 pathway [31]. These studies break the traditional view that lactate is a waste product of glycolysis and indicate that lactate may play an important role as a signaling molecule in the CNS. Several studies have examined the role of lactate as a signaling molecule involved in the regulation of the physiological function of astrocytes and neurons [30, 32–37]. Deeper look into the effect and mechanism of exercise-induced lactate on specific cells types in the brain in neurodegenerative disease progression is still required.

Metabolites can induce post-translational modifications of histones and regulate gene transcription and expression by altering chromatin structure, including lactate-induced lactylation (Kla) [38]. Recently, a novel theory called the “lactate timer” has been proposed, this theory demonstrated that elevated levels of lactate in inflammatory stimulated macrophages directly acts on Histone H3s, H3K18la-specific genes were enriched in steady state and repairing gene promoter to promote the expression of homeostatic genes involved in the process of damage repair. Enrichment of Kla and increased expression of the Arginase 1 (Arg1, a marker of reparative phenotype) in macrophages was observed when treated with exogenous lactate [39]. Therefore, lactate act as a “timer” in the progress of phenotype transition from pro-inflammatory to anti-inflammatory/reparative. Since microglia are also phagocytic cells it is possible that the elevated lactate levels induced by exercise may act as an accelerator of a “lactate timer” in microglia through regulation of epigenetic modifications, and thus induce the reparative phenotype transition of microglia in AD.

D-galactose (D-gal) is a senescence agent, while aluminium is a known neurotoxin linked to pathogenesis of AD. There is ample evidence that D-gal/aluminum chloride (D-gal/AlCl_3_) combined treatment can induce AD-like symptoms, including cognitive and memory impairments, overexpression of amyloidogenic proteins, oxidative damage, microglia activation and neuroinflammation [40–44]. In addition, aging is one of the risk factors of neurodegenerative diseases [45, 46]. In this study, We have established D-gal and AlCl_3_-induced AD-like mouse model and aging mouse models of different ages, and demonstrate that an exercise-induced elevation in lactate in the brain of mice acts as an endogenous accelerator of the “lactate timer” in brain microglia. We show that exercise amplifies the transition of microglia from a damaging to a reparative phenotype through Kla of microglia Histones H3 and that this improves neurological and cognitive function in mouse models. Finally, we demonstrate that lactate addition to cultured BV2 microglia cells accelerates this transition.

## Results

### Exercise improves cognitive function of AlCl_3_/D-gal-treated mice and aging mice

**Fig. 1:**
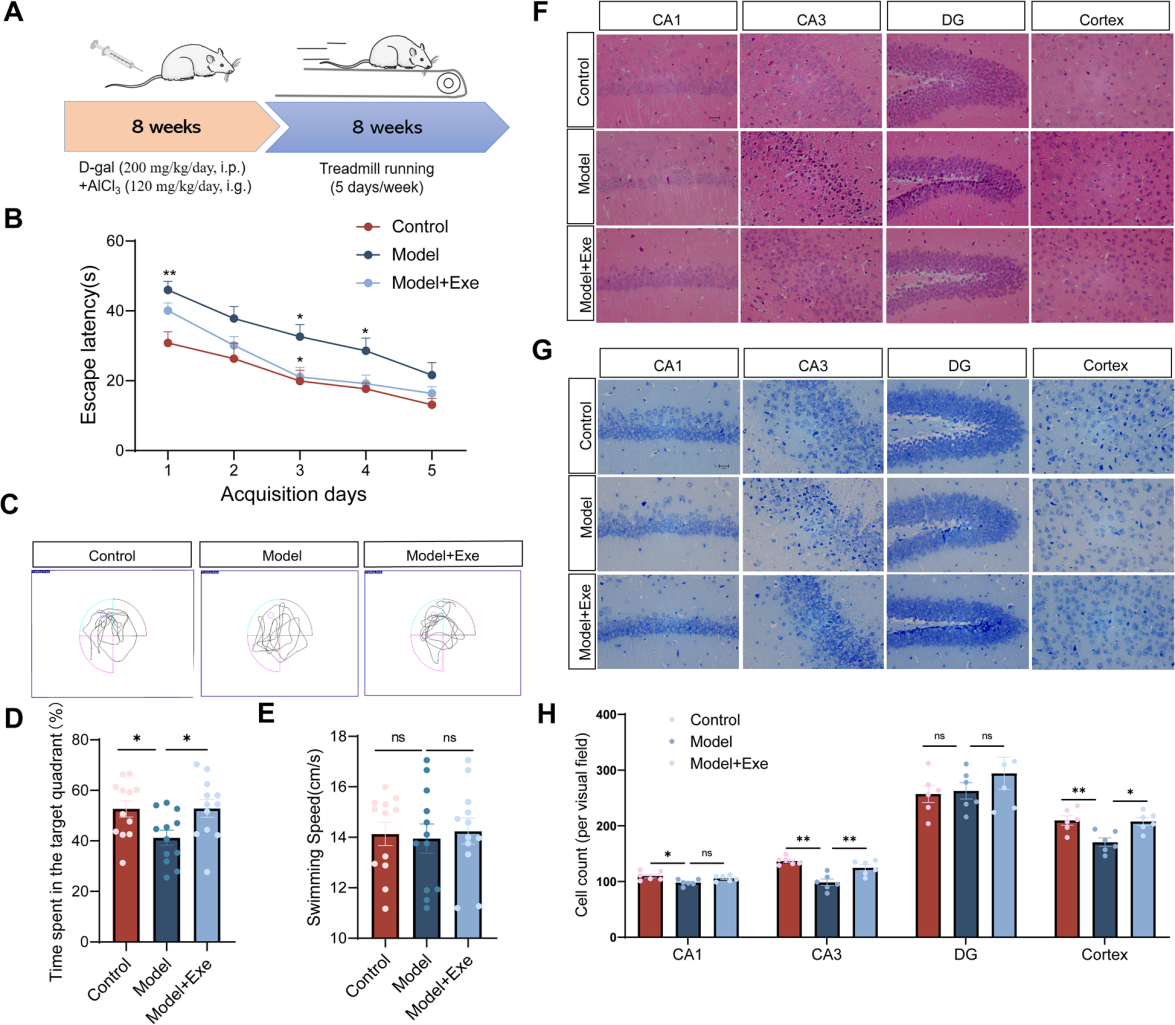
Exercise improves cognitive function and reverses hippocampal neuron loss in AlCl_3_/D-gal-treated mice. **A** Flow chart for the experimental design. **B** The escape latency of mice to the platform during the acquisition phase (*n* = 12 per group). **C** Representative track images of each group mice in day 6 probe trial test. **D** Time spent in the target quadrant (%) within 60 s of mice in the probe trial (*n* = 12 per group). **E** The mean swimming speed of each group mice in day 6 probe trial test (*n* = 12 per group). **F** Representative images of HE staining in various brain regions (magnification 400 ×). **G** Representative images of Nissl staining in various brain regions (magnification 400 ×). **H** The number of living neurons of hippocampus and prefrontal cortex in mice (*n* = 6 per group). Data are means ± SEM. *p < 0.05, **p < 0.01 *vs*. Control group; *p < 0.05 in *vs*. Model group in B. *p < 0.05, **p < 0.01, ns-not significant in D, E and H. Statistical analysis was performed using two-way (**B**) and or one-way (**D**, **E**, **H**) ANOVA, followed by Tukey’s multiple comparisons test

To investigate the effect of exercise on the cognitive function of mice, we prepared AD-like model mice using D-gal and AlCl_3_ for in vivo studies and combined this with moderate-intensity treadmill running exercise intervention (Fig. 1A). After exercise training, the Morris water maze test was used to evaluate the cognitive function of mice. Learning and memory ability was significant attenuated in AlCl_3_/D-gal-treated mice (Model) compared to control (Control) as indicated by longer escape latency and decreased target quadrant occupation. In contrast, learning and memory ability was markedly improved in AlCl_3_/D-gal-treated mice after exercise training (Model + Exe) (Fig. 1B-D). No significant difference in the swimming speed in Morris water maze test was observed among all groups indicating that time spent in escape latency was not effected by differences in swimming speed between groups (Fig. 1E). We investigated the morphology and numbers of neurons using HE and Nissl staining. In the Control mice, cells in the hippocampal region showed an orderly arrangement with no apparent abnormalities in cellular morphology. Nissl bodies were clearly visible and evenly distributed in the cytoplasm. In contrast, neuronal loss and neurodegeneration was observed in the hippocampus and cortex of Model mice. Exercise training appeared to attenuate this in Model + Exe mice (Fig. 1F-H).

**Fig. 2:**
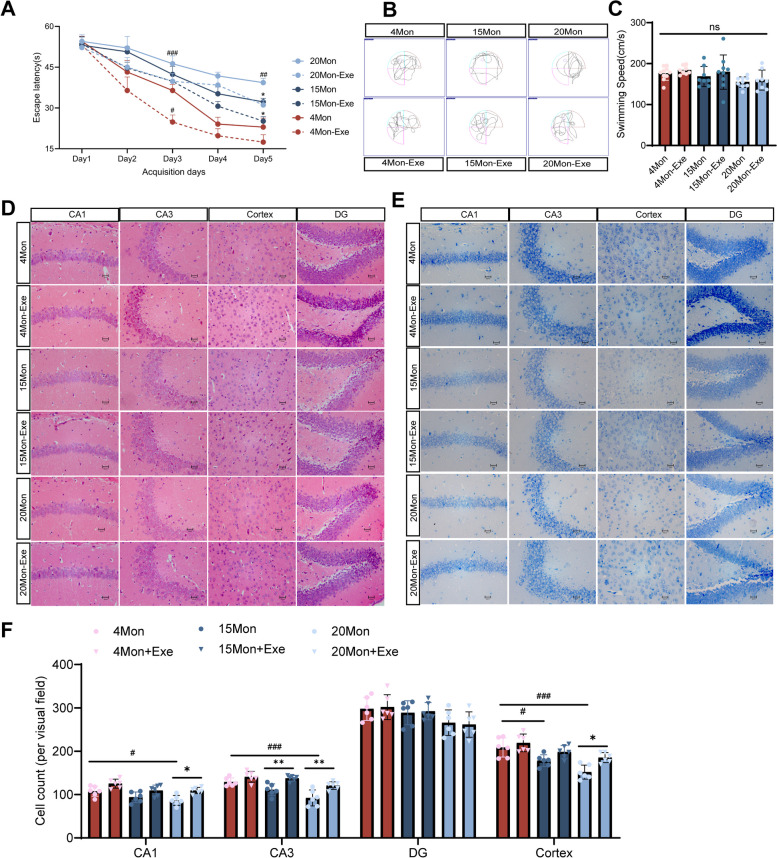
Exercise improves cognitive function and reverses hippocampal neuron loss in aging mice. **A** The escape latency of mice to the platform during the acquisition phase. (*n* = 8–10 per group). **B** Representative track images of each group mice in day 6 probe trial test. **C** The mean swimming speed of each group mice in day 6 probe trial test (*n* = 8–10 per group). **D** Representative images of HE staining in various brain regions (magnification 400 ×). **E** Representative images of Nissl staining in various brain regions (magnification 400 ×). **F** The number of living neurons of hippocampus and prefrontal cortex in mice (*n* = 6 per group). Data are means ± SD. ^#^*p* < 0.05, ^##^*p* < 0.01, ^*###*^*p* < 0.001 vs. 4 Mon group; *p < 0.05, **p < 0.01 in vs. 15 Mon group; *p < 0.05, **p < 0.01 in vs. 20 Mon group in A and F; ns-not significant in C. Statistical analysis was performed using two-way (**A**) and or one-way (**C**, **F**) ANOVA, followed by Tukey’s multiple comparisons test

Since aging is a common contributor to development of AD, we evaluated the cognitive function and the effect of exercise in young and aged mice. Mice of different age (4, 15 and 20 months) were used as representative of young, middle aged and old. Considering the difference in running ability between young and old mice, the maximum running speed test was recorded each week before the weekly running exercise training, and 70% of the average maximum running speed of each group was used as their weekly running speed [47, 48]. As expected, maximal running speed increased rapidly in the first few weeks, stabilized in the following weeks, demonstrating that older mice have slightly lower maximum running speed than younger mice (Fig. S1). Learning and memory function was then assessed by Morris water maze. We observed that 20-month-old mice exhibited a decrease learning and memory abilities compared to 4-month-old mice. Exercise training significantly improved the escape latency in 4-month-old, 15-month-old and 20-month-old mice (Fig. 2A-B) and had no effect on swimming speed (Fig. 2C). But the time spent in the target quadrant within 60 s did not differ significantly between the groups of mice in the probe test, most likely due to the small number (data not shown). These results indicate that learning and memory abilities may reduce with age and the improvement of these by exercise training may be more effective in both young and old mice. Increasing age of the mice resulted in neurodegeneration and neuronal loss in the hippocampal and in the prefrontal cortex area of 15-month-old and 20-month-old mice compared to 4-month-old mice. In contrast, after 8 weeks of running exercise training the abnormal morphology and loss of hippocampal neurons was attenuated in these mice (Fig. 2D-E).

### Exercise increases the reparative microglia in AlCl_3_/D-gal-treated mice

**Fig. 3:**
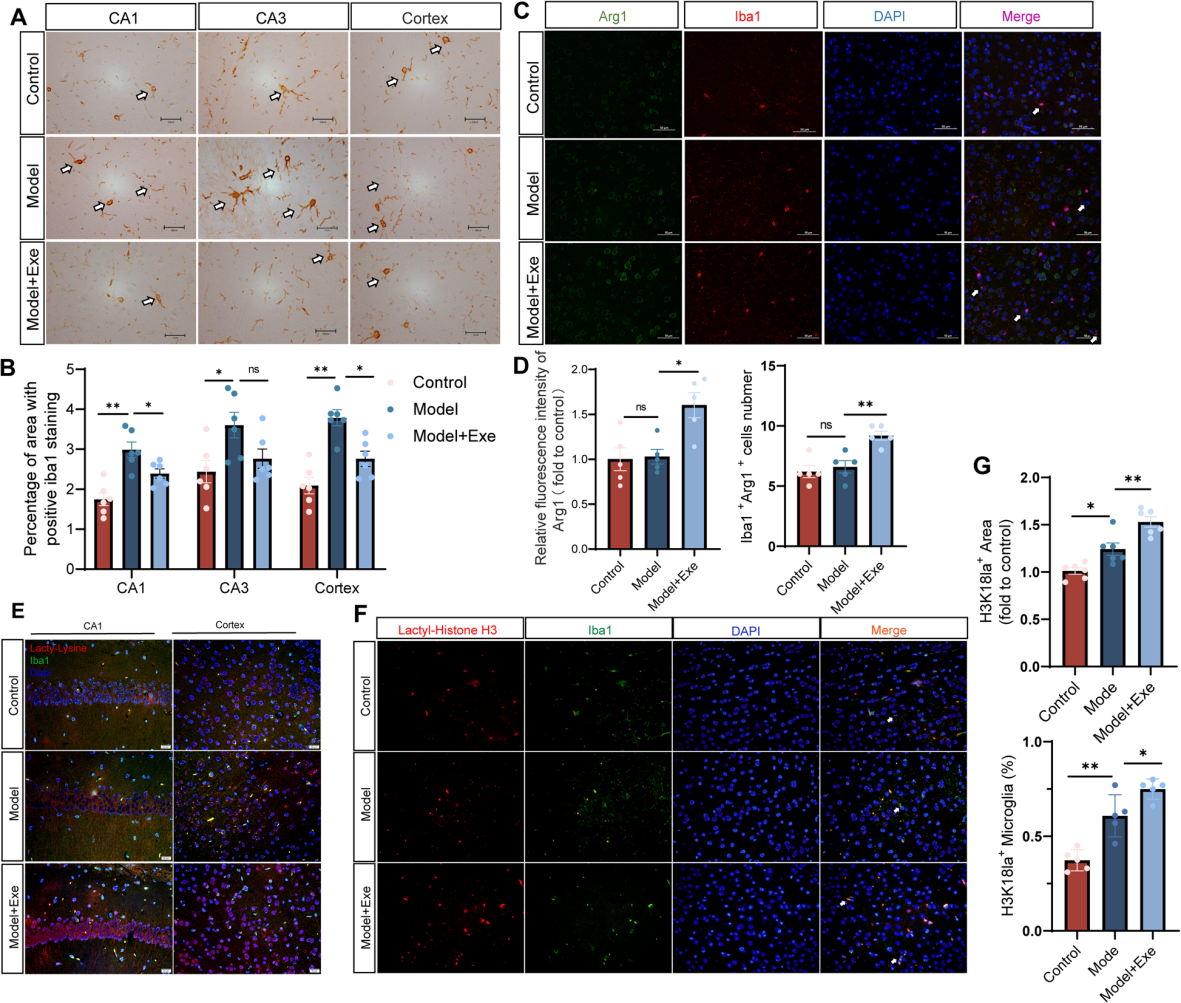
Running exercise modulates microglia polarization phenotype in AlCl_3_/D-gal-treated mice. **A** Representative images of Iba1 staining in various brain regions (magnification 400 ×). **B** Quantification of area covered by Iba1^+^ cells per image (*n* = 6 per group). **C** Double immunofluorescent staining of Arg1 (green) and Iba1 (red) of mice in each group (magnification 400 ×), arrows indicate Arg 1^+^ Iba 1^+^ cells. **D** Quantification of Arg1 Fluorescence intensity (normalized to Control group) and number of Iba1^+^Arg1^+^ cells (*n* = 5 per group). **E** Double immunofluorescent staining of iba1 (green) and Pan-Kla (red) of mice in each group (magnification 400 ×). **F** Double immunofluorescent staining of Iba1 (green) and H3K18la (red) of mice in each group (magnification 400 × , arrows indicate H3K18la^+^ Iba1^+^ cells). **G** Quantification of area covered by H3K18la^+^ per image (*n* = 6 per group) and the ratio of H3K18la^+^ Iba1^+^ cells/Iba1^+^ cells (*n* = 5 per group). Data are means ± SEM. *p < 0.05, **p < 0.01, ns-not significant in **B**, **D** and **G**. Statistical analysis was performed using one-way ANOVA, followed by Tukey’s multiple comparisons test

Microglia play an important role in AD development and reparative microglia may promote neuroprotection in AD [49, 50]. Iba1, a microglia marker, was increased in the hippocampus and cortex regions of AD mice (Model) compared to control (Control). Compared with the mice in the Model group, the Model + Exe group mice showed reduced iba1 expression in the hippocampal CA1 region and cortex (Fig. 3A-B). Expression of Arg1, a marker of reparative microglia, and co-localization of iba1 with Arg1 was then performed [51, 52]. Arg1 expression was unaltered between Control and Model mice but was increased in Model + Exe mice compared to Model mice. Furthermore, there was no difference in the number of Arg1-positive microglia between Control mice and Model mice. In contrast, the number of Arg1-positive microglia was increased in Model + Exe mice compared to Model mice (Fig. 3C-D). These data indicate that exercise training increased reparative microglia in AD-like mice.

### Exercise induces Histone H3 lactylation in the brain of AlCl_3_/D-gal-treated mice and aging mice

Lactate is a major product of exercise and is transported by blood to various tissues throughout the body [53, 54]. In addition, lactate levels are elevated post running exercise [26]. We confirmed that lactate levels were increased in both plasma and brain of mice subjected to exercise training (Fig. S2A-B). Lactate serves not only as an energy source for brain cells but also as a signaling molecule through lactylation [31, 55, 56]. Therefore, we analyzed lactylation and Histone H3 Kla in the brain of AlCl_3_/D-gal-treated mice and aging mice. Brain lactylation and Histone H3 Kla was increased in Model mice compared to Control mice and was further elevated after running exercise in Model + Exe mice (Fig. 3E-G). In addition, brain Histone H3 Kla was increased by exercise not only in young but in all ages of mice (Fig. S3A-B). This indicated exercise induces Histone H3 Kla in the brain of AD and in the brain of aging mice.

### Sodium lactate injection improves cognitive function in AlCl_3_/D-gal-treated mice

**Fig. 4:**
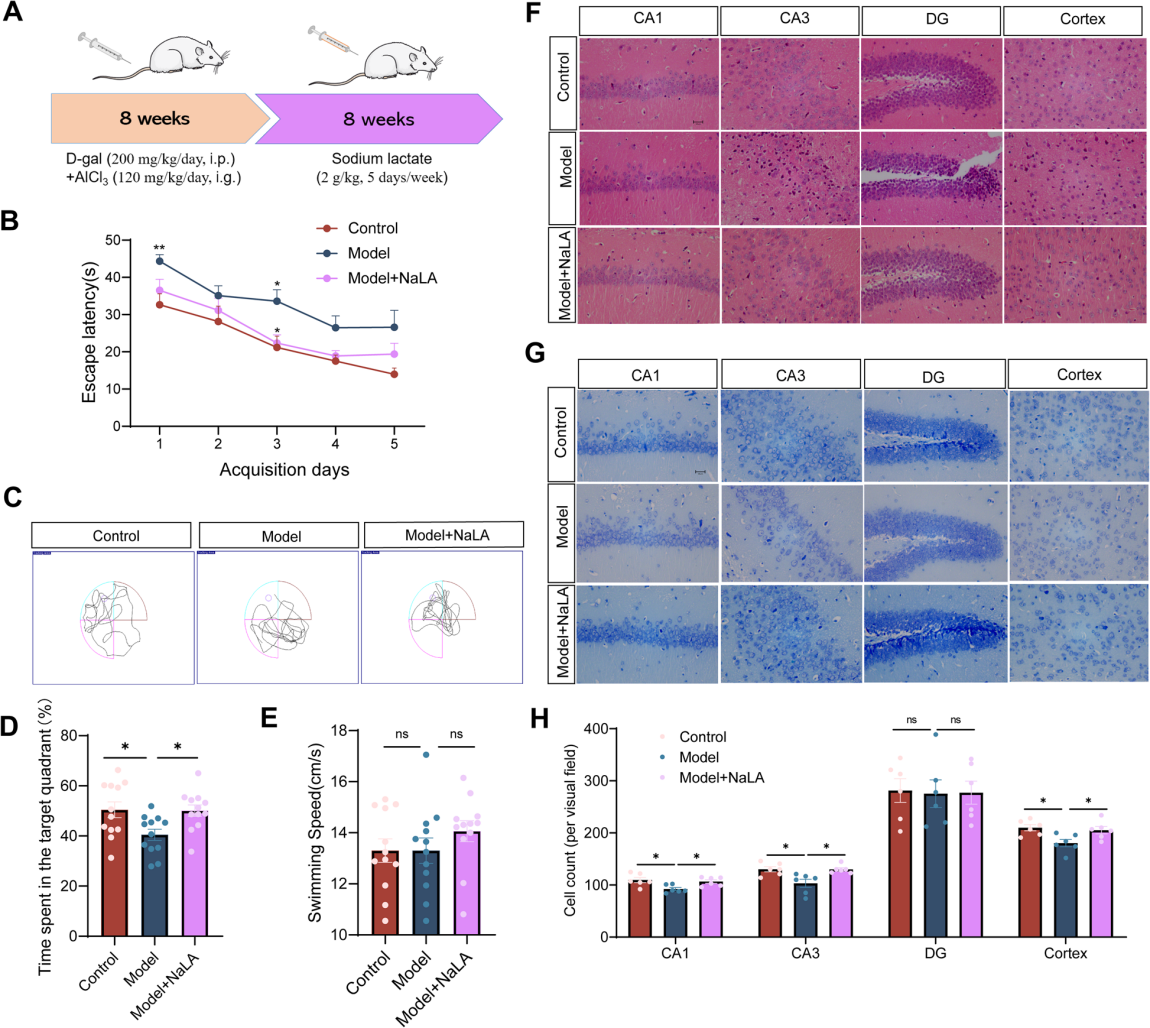
Sodium lactate injection improves cognitive function and reverses hippocampal neuron loss in AlCl_3_/D-gal-treated mice. **A** Flow chart for the experimental design. **B** The escape latency of mice to the platform during the acquisition phase. (*n* = 12 per group). **C** Representative track images of each group mice in day 6 probe trial test. **D** Time spent in the target quadrant (%) within 60 s of mice in the probe trial (*n* = 12 per group). **E** The mean swimming speed of each group mice in day 6 probe trial test (*n* = 12 per group). **F** Representative images of HE staining in various brain regions (magnification 400 ×). **G** Representative images of Nissl staining in various brain regions (magnification 400 ×). **H** The number of living neurons of hippocampus and prefrontal cortex in mice (*n* = 6 per group). Data are means ± SEM. *p < 0.05, **p < 0.01 vs. Control group; *p < 0.05 in vs. Model group in B. *p < 0.05, ns-not significant in D, E and H. Statistical analysis was performed using two-way (**B**) and or one-way (**D**, **E**, **H**) ANOVA, followed by Tukey’s multiple comparisons test

To demonstrate whether the cognitive function improvement after exercise in mice was mediated by lactate, exogenous sodium lactate (NaLA) was intraperitoneally injected into the AlCl_3_/D-gal-treated mice for up to 8 weeks and cognitive function examined using Morris water maze analysis (Fig. 4A). Plasma and brain lactate levels were significantly increased in Model mice after sodium lactate treatment (Fig. S2C-D). Learning and memory ability was significantly improved in Model + NaLA mice compared to Model mice and was restored to Control values (Fig. 4B-D). NaLA injection treatment did not affect the swimming speed of mice during the Morris water maze text (Fig. 4E). HE staining and Nissl staining indicated that exogenous lactate reversed neuronal loss and neurodegeneration in Model + NaLA mice compared to Model mice (Fig. 4F-H), suggesting that exogenous lactate treatment can have beneficial effects similar to exercise in mice.

### Lactate treatment induces Histone H3 lactylation and promotes expression of the reparative phenotype of microglia in AlCl_3_/D-gal-treated mice

During aging and AD progression, the transition of oxidative phosphorylation (OXPHOS) to glycolysis in the activation of microglia accompanies an increase in lactate levels in the brain [57, 58], which may turn on the “lactate timer” in microglia to maintain a homeostatic environment. We speculate that the increased Kla observed in AlCl_3_/D-gal-treated mice and aging mice may be due to an endogenous feedback regulatory mechanism against the nerve damage and Aβ deposition. However, this endogenous feedback regulatory mechanism was not sufficient to elevate Histone H3 Kla to a level that would promote generation of reparative microglia nor reverse existing neuronal damage in our AD mice. Given that the level of Histone H3 Kla was elevated in AD-like mice and exercise-trained mice (Fig. 3E-G, Fig. S3A-B), it is possible that lactate may serve as a contributor to improved cognitive function and reparative microglia phenotype in mice and that the exercise-induced increase in lactate may act as an ‘accelerator’ of the ‘lactate timer’ to amplify the reparative effect of microglia.

**Fig. 5:**
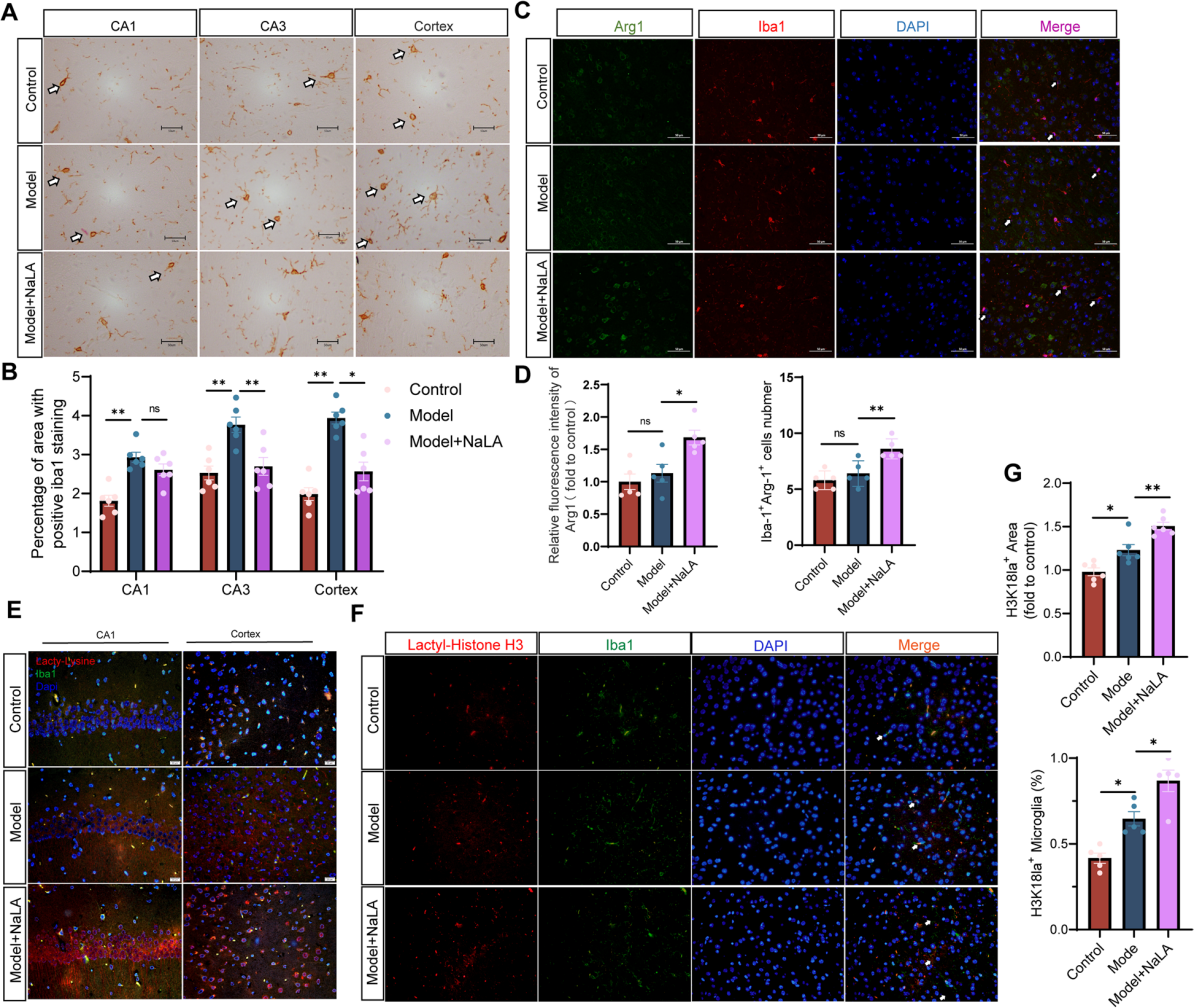
Sodium lactate injection modulates microglia polarization phenotype in AlCl_3_/D-gal-treated mice. **A** Representative images of Iba1 staining in various brain regions (magnification 400 ×). **B** Quantification of area covered by Iba1^+^ cells per image (*n* = 6 per group). **C** Double immunofluorescent staining of Arg1 (green) and Iba1 (red) of mice in each group (magnification 400 ×), arrows indicate Arg 1^+^ Iba 1^+^ cells. **D** Quantification of Arg1 Fluorescence intensity (normalized to Control group) and number of Iba1^+^Arg1^+^ cells (*n* = 5 per group). **E** Double immunofluorescent staining of iba1 (green) and Pan-Kla (red) of mice in each group (magnification 400 ×). **F** Double immunofluorescent staining of Iba1 (green) and H3K18la (red) of mice in each group (magnification 400 × , arrows indicate H3K18la^+^ Iba1^+^ cells). **G** Quantification of area covered by H3K18la^+^ per image (*n* = 6 per group) and the ratio of H3K18la^+^ Iba1^+^ cells/Iba1^+^ cells (*n* = 5 per group). Data are means ± SEM. *p < 0.05, **p < 0.01, ns-not significant in B, D and G. Statistical analysis was performed using one-way ANOVA, followed by Tukey’s multiple comparisons test

We thus examined the impact of exogenous lactate treatment on the exacerbated microglia activation in AlCl_3_/D-gal-treated mice. The results show that sodium lactate treatment reverses increased microglial activation in Model mice (Fig. 5A-B). In addition, sodium lactate treatment significantly induced Arg1 expression in Model + NaLA mice compared to Model mice and thus increased the number of reparative microglia (Fig. 5C-D). The Kla of Histone H3 in microglia were then analyzed, as expected, exogenous sodium lactate increased the Histone H3 Kla level of microglia in AlCl_3_/D-gal-treated mice (Fig. 5E-G). Taken together, these data indicate that lactate induced the Histone H3 Kla which promotes expression of the reparative phenotype of microglia in mice.

### Lactate treatment inhibits pro-inflammatory and enhances anti-inflammatory gene expression in activated BV2 microglia cells

**Fig. 6:**
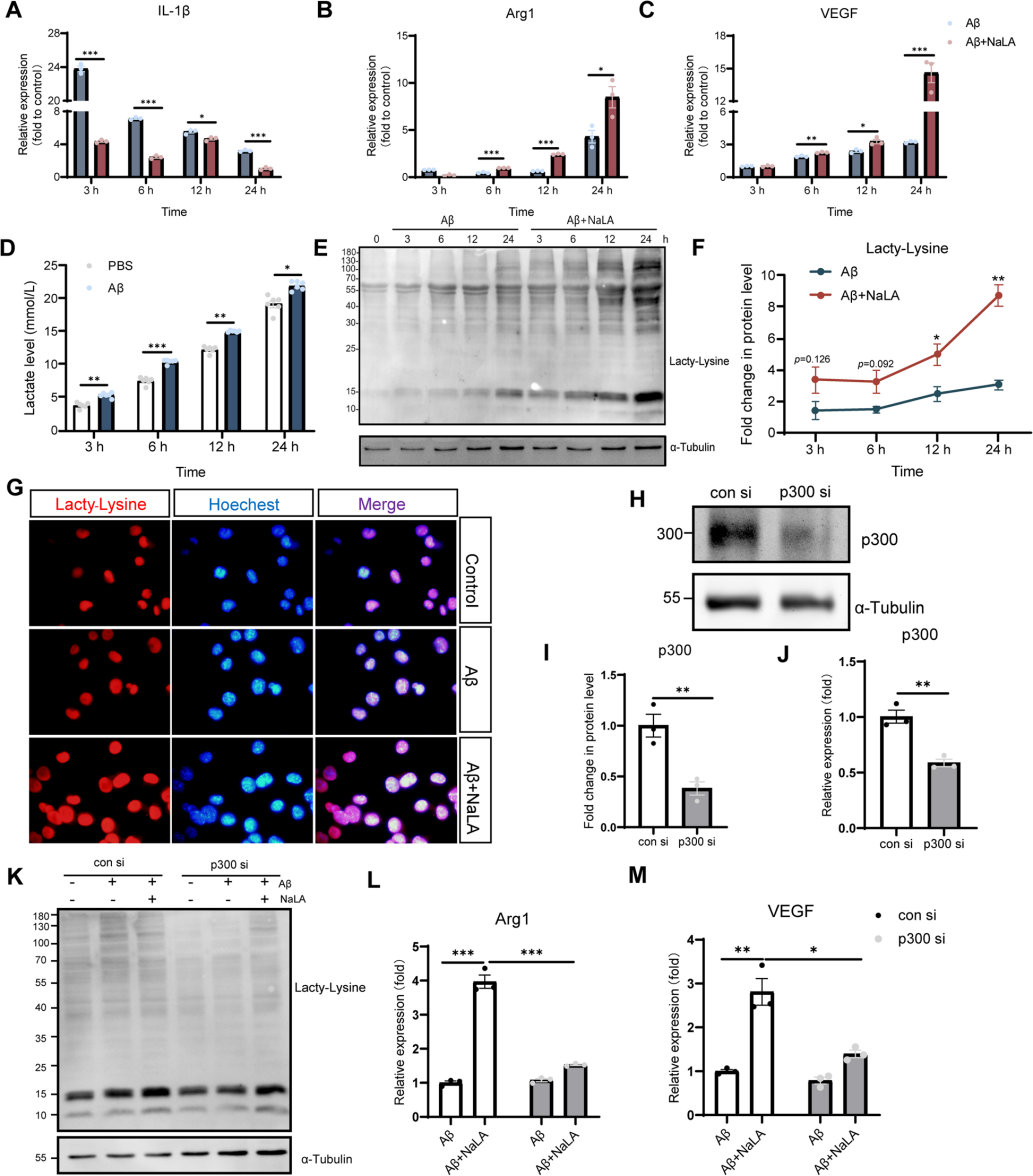
Lactate regulates BV2 cell phenotype through lactylation. **A**-**C** Expression of IL-1β (**A**), Arg1 (**B**), and VEGF (**C**) at different time points in BV2 cells incubated with Aβ_1-42_ and Aβ_1-42_ + NaLA (*n* = 3 per group, normalized to the untreated sample). **D** Lactate levels measured at different time points in the conditioned medium of BV2 cells treated with PBS or Aβ_1-42_(*n* = 6 per group). **E**–**F** Western blot shows the levels of Histone Kla in BV2 cells incubated with Aβ_1-42_ or Aβ_1-42_ + NaLA at different time points (*n* = 3 per group, normalized to expression at 0 h). **G** Representative images of Pan-Kla immunofluorescence staining. (H-I) Representative western blot and western blot analysis of p300 in BV2 cells (*n* = 3 per group). **J** The mRNA expression of p300 (*n* = 3 per group). **K** Representative images of Pan Kla western bolts in BV2 cells transfected with con siRNA or p300 si RNA. **L**-**M** Expression of related mRNA in BV2 cells transfected with con si RNA or p300 si RNA (*n* = 3 per group). Date are means ± SEM. *p < 0.05, **p < 0.01, ***p < 0.001. Statistical analysis was performed using unpaired Student’s two-tailed t-tests (**A**-**D**, **F**, **I**-**J**) or one-way ANOVA, followed by Tukey’s multiple comparisons test (**I**-**M**)

To further understand the role of lactate-induced Kla in promotion of the reparative microglia phenotype, we incubated the microglia cell line BV2 with Aβ_1-42_ to promote activation and examined mRNA expression of the pro-inflammatory gene IL-1β and the repair genes Arg1 and VEGF. As expected, expression of IL-1β gene increased sharply at the initial stage of Aβ_1-42_ inflammatory stimulation, and then gradually decreased over time. In addition, we observed that Arg1 and VEGF were increased in the late stage of BV2 activation, which indicated that microglia gradually shifted to a repair phenotype after activation (Fig. 6A-C). However, the spontaneously feedback activation of repair genes was mild especially at the early stage after stimulation, thereby not able to alter the pro-inflammatory state of the microglia. Addition of NaLA to Aβ_1-42_-stimulated cultures reduced IL-1β expression at all time points tested. Meanwhile, Arg1 and VEGF expressions were significantly increased in Aβ_1-42_-stimulated BV2 cells after treatment with sodium lactate for 6 h and at various time points thereafter compared with untreated cells (Fig. 6A-C). Similarly, we stimulated BV2 cells with LPS for 24 h to promote activation, and added NaLA to the medium for 24 h. We observed that the mRNA expression of Arg1 and VEGF was also significantly increased in LPS-stimulated BV2 cells after the addition of NaLA (Fig. S4A-B). These results may imply that sodium lactate accelerated the transition of activated BV2 cells to an anti-inflammatory/reparative phenotype.

We hypothesized that the changes in gene expression levels observed in Aβ_1-42_/LPS-stimulated BV2 cells depended on microglia plasticity, and lactate-induced Kla play an important role in mediating the shift of activated BV2 cells towards an anti-inflammatory/reparative phenotype. As expected, under the stimulation of Aβ_1-42_, BV2 cells secrete more lactic acid (Fig. 6D). Consistent with this, we observed that Histone H3 Kla levels gradually increased over time in Aβ_1-42_-stimulated BV2 cells by western bolt analysis using Pan Kla antibody. After adding sodium lactate to Aβ_1-42_-treated BV2 cells, the Kla level of BV2 cells was significantly increased compared with cells treated with Aβ_1-42_ (Fig. 6E-G). Furthermore, a similar phenomenon was observed in LPS-stimulated BV2 (Fig. S4C-D**)**. According to reports, the acetyltransferase p300 has been implicated in the histone Kla [39, 59, 60]. We knocked down p300 with siRNA (Fig. 6H-J) and observed that the p300 knocking down reversed the Kla induced by Aβ_1-42_ and sodium lactate (Fig. 6K). Further, it was observed that that the induction of the Arg1 and VEGF expression by sodium lactate was dampened when p300 was knocked down in these cells (Fig. 6L-M). The above experiments show that, mediated by p300, lactate-induced Kla induces the expression of anti-inflammatory/repair genes in BV2 cells.

**Fig. 7:**
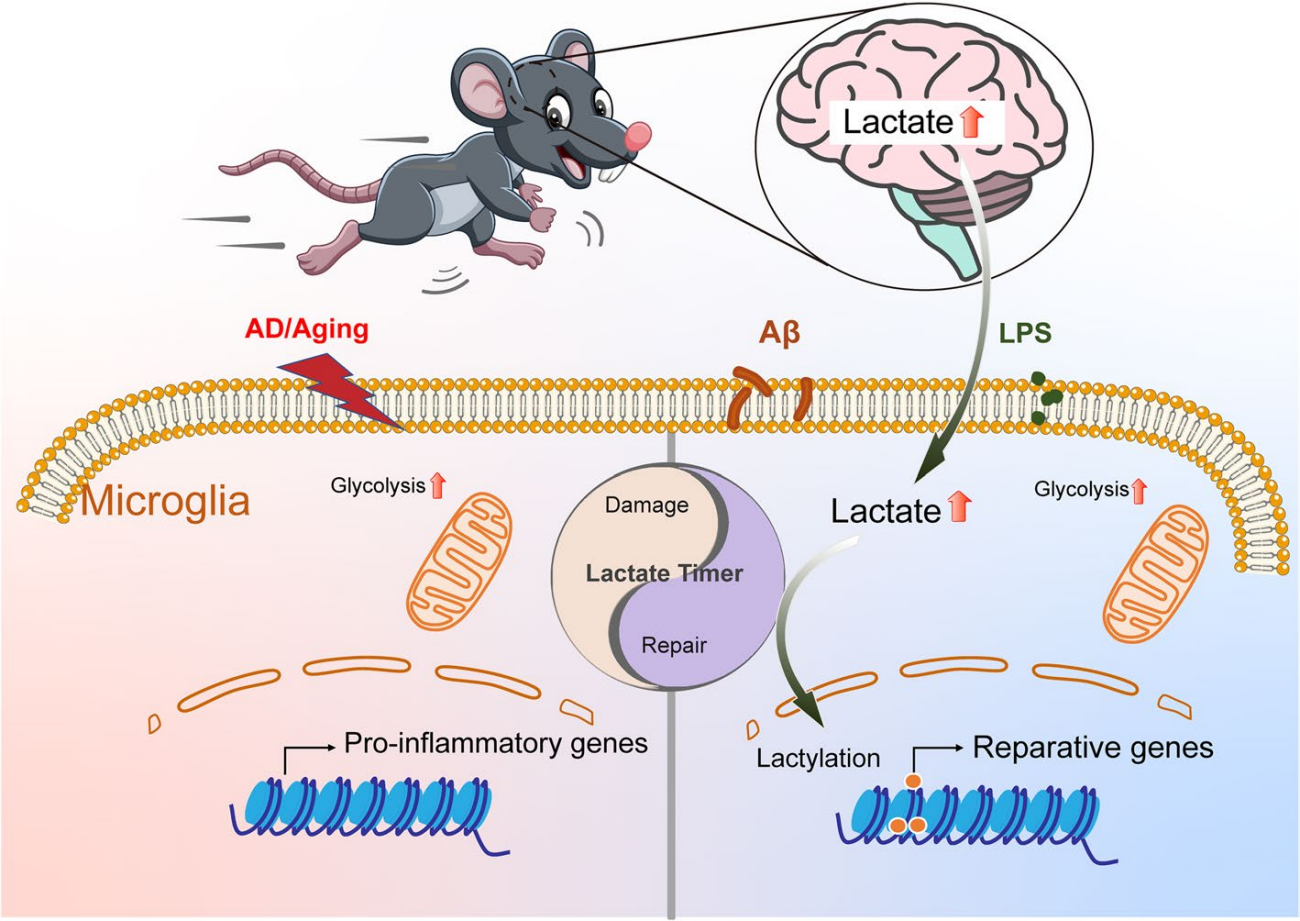
Graphical abstract. Exercise or lactate injection acts as an “accelerator” for the endogenous “lactate timer” of microglia, and improve cognitive function by regulating the anti-/pro-inflammatory balance of microglia in AD progression

In conclusion, our results indicate that exogenous lactate treatment promotes transition of pro-inflammatory microglia to anti-inflammatory/reparative phenotype. Exercise training or lactate injection may function as an “accelerator” for the endogenous “lactate timer” of microglia, to improve cognitive function by regulating the anti-inflammatory/pro-inflammatory balance of microglia in the process of AD (Fig. 7).

## Discussion

Lifestyle changes including increased physical activity is an effective strategy for delaying the progression of neurodegenerative disease [61, 62]. Several studies have proposed a possible link between exercise training and cognitive improvement [15, 63, 64]. We trained mice to run at increasing speed over 8 weeks which represents a typical in vivo model of physical activity [30, 65–68]. The principal findings of our study are 1. Exercise training improves cognitive function in AlCl_3_/D-gal-treated mice and aging mice by reducing neuronal loss and neuroinflammation, and 2. Elevated levels of lactate in the brain attenuate this neuroinflammation by acting as an “accelerator” for the “lactate timer” in microglia by promoting transition to a reparative phenotype through Histone H3 Kla. Our results provide an extension to the beneficial effects of exercise training beyond strengthening skeletal muscle, and further confirm that exercise training improves cognitive function and reverses neuronal loss in the brain of AD-like mice.

Previous studies have demonstrated that exercise increases metabolic factors and muscle-derived cytokines that help prevent AD risk and progression [12]. Lactate is a major exercise-induced products [69, 70]. We identified lactate as a novel endogenous metabolite that links exercise and cognition improvement. We observed that post-exercise training of mice exhibited increased levels of lactate in the brain and plasma and this accompanied improvement in cognition. In addition, when AD-like mice were injected with exogenous lactate the concentration of circulating lactate levels were elevated to similar levels observed after exercise training and this accompanied improvement in cognition. Thus, exogenous lactate improves the cognitive function of mice similar to that of exercise training and confirms a crucial role of lactate in exercise-induced cognition improvement.

Although elevated lactate is observed in the brain after exercise training it is currently unknown how exercise training promotes an increase in lactate levels in this organ. In skeletal muscle cells ATP is mainly generated from glucose to fuel glycolysis during vigorous exercise. Lactate, as product of glycolysis, is then transported through the circulation and entered into the brain blood brain barrier transporters belonging to the monocarboxylate carrier (MCT) family [28, 71, 72]. Studies of whole-brain metabolic activity during exercise revealed progressive increases in brain lactate uptake and metabolism as workload and plasma lactate levels increase [73, 74]. However, the brain itself may generate lactate. Brain lactate release is approximately 0.05 mmol·min^−1^ at rest and doubles during exercise [75, 76], The lactate released from the brain is primarily from astrocytes and serves as fuel for neighboring cells [77–79]. Although our study did not identify the source of the elevated brain lactate during exercise training we confirmed that exercise training elevated brain lactate levels. Further studies on the source of exercise-induced brain lactate are warranted.

Other studies have attributed the beneficial effects of exercise to lactate. For example, lactate partially mediates the effect of physical exercise on neurogenesis in a MCT2-dependent manner [80]. Subcutaneous injection of lactate lead to an increase in blood lactate levels similar to exercise and increases brain VEGF protein [30]. These studies provide a preliminary link between exercise, lactate, and cognitive function. Although studies demonstrated an important role of lactate in physiological function in neurons and astrocytes, there has been little empirical investigation on microglia. Over the past thirty years, microglia are traditionally described as two states, resting and activated [81]. The reactive gliosis observed in AD histopathology reflects an abnormal morphology and proliferation of microglia [82]. Once overactivated microglia release a wide range of inflammatory and bioactive molecules which impose negative impacts on neurons [7]. Extensive activation of microglia was detected in our AD mice and may contribute to the observed cognitive impairment. It is now recognized that the activated microglia can broadly exist in two distinct states [83]. One is classical activation typified by the production of pro-inflammatory cytokines and reactive oxygen species (ROS) while the other is alternative activation in which microglia take on an anti-inflammatory/reparative phenotype involved in wound repair and debris clearance [84]. These two states in the activated microglia is constantly changing and reaching a balance in the brain environment. In AD patients, the alternative activation of microglia appears to be inhibited favoring classical activation [85]. This may be the reason why, despite the massive activation of microglia in our AD-like mice, the number of anti-inflammatory/reparative microglia did not increase. However, both running training and exogenous lactate treatment inhibited the hyperactivation of microglia in AD-like mice and increased the number of anti-inflammatory/reparative microglia. In vitro experiments in BV2 microglia confirmed that lactate treatment significantly increases the expression of repair genes, indicating that lactate may promote a shift in balance from damaging to reparative microglia.

The concept of “lactate timer” was first proposed in the study of macrophages [39]. Zhang et al. reported an increased aerobic glycolysis leading to enhanced lactate production during M1 (inflammatory) macrophage polarization, thereby triggering the activation of the ‘lactate timer’ mechanism. This timer induces M2 (anti-inflammatory/reparative) gene expression at the late stage of M1-type macrophage polarization by mediating the Kla modification of Histone H3 lysines [39, 86]. Exogenous lactate was shown to induce the M2 phenotype of macrophages and participate in neovascularization by promoting the expression of VEGF in macrophages [87, 88]. In addition, exogenous lactate restored homeostatic gene expression in BCAP-deficient macrophages by inducing Kla [89]. In pathological conditions of AD brain glucose metabolism is altered [90]. Like macrophages, microglia activation is accompanied by extensive transcriptional changes in genes involved in glucose and lipid metabolism, characterized by repression of oxidative phosphorylation and activation of glycolysis [57, 91]. We found that Histone H3 Kla was significantly increased in AD-like mice compared to normal mice and that Histone H3 Kla was gradually induced with age. In BV2 microglia stimulated by Aβ_1-42_, the expression of pro-inflammatory gene IL-1β was decreased and the expression of repair gene Arg1 and VEGF were increased temporally with Kla. These results indicated that a “lactate timer” may also exist in microglia as part of brain’s self-repairing mechanism regulating the expression of repair genes to maintain a homeostatic environment. However, this self-repairing mechanism may be insufficient on its own to rescue excessive microglia activation in AD. In our study, we observed that exercise training or lactate treatment further increased the level of lactate and the reparative microglia in the brain of AD-like mice. However, our research has certain limitations. Firstly, we cannot rule out the influence of sex factors on the findings, and we only used male mice, which limits the interpretation of our results. Secondly, in our study, we only used BV2 cells for in vitro experiments and did not conduct experiments with age-matched primary microglia to determine whether similar results could be obtained. Future research directions may involve conducting in vitro experiments on primary microglia derived from AD patients or aging mice to further investigate the effects of lactylation under these specific physiological conditions.

In summary, these findings suggest that exercise-induced lactate levels in the brain may act as an “accelerator” for the “lactate timer” of microglia amplifying a reparative microglia transition through lactate modification to maintain brain homeostasis.

## Methods

### Animals

In this study, D-gal/AlCl_3_-induced AD-like mice and mice of different ages were used to study. All mice were purchased from Skbex Biotechnology Co., Ltd. [SCXK (Yu) 2020–0005]. The mice were housed in a room with suitable temperature and maintained on a 12 h day/night cycle. For AD-like mice, the 5–6-week-old male C57BL/6 J mice (*n* = 90) were randomly assigned into four groups and treated daily for eight weeks as follows; One group of mice (Control, *n* = 30) served as the control (Control) and saline was administered either orally and through intra-peritoneal injection (i.p). The other three groups of mice were injected i.p with D-gal 200 mg/kg body weight and orally administered AlCl_3_ 100 mg/kg body weight for 8 weeks to promote AD model development [92, 93]. After 8 weeks, these mice were divided as follows: untreated (Model, *n* = 20), running exercise training (Model + Exe, *n* = 20) and i.p. sodium lactate injection (Model + NaLA, *n* = 20). Mice were regularly observed weekly and body weight changes were recorded. For the mice of different ages, three different age groups (young, 4 months, *n* = 20; middle-aged, 15 months, *n* = 20; and old, 20 months, *n* = 20), were used for the study. Mice were paired for normal activity or running training for each age group (4 Mon/4 Mon-Exe, 15 Mon/ 15 Mon-Exe, 20 Mon/ 20 Mon-Exe). This study is reported in accordance with ARRIVE guidelines. All animal experimental procedures were approved by the Ethics Committee for the Use of Experimental Animals of Jilin University [SYXK (Ji) 2018–0001].

### Exercise training protocol

Exercise training was performed on a mouse treadmill (Taimeng, Chengdu, China). For AlCl_3_/D-gal-treated mice three days of treadmill adaptive training was carried out. Specifically, the treadmill was set at an incline of 0° for all activities, running training was a speed of 8 m/min for 10 min on the first day; 12 m/min on the second day for 10 min; and on the third day 16 m/min for 10 min. Formal running training was started on the fourth day. The training program was as follows: the mice first performed a 10-min warm-up exercise at a running speed of 10 m/min and then the speed was adjusted to 16 m/min and they continued to run for 40 min. The running speed was increased by 1 m/min every week for 8 weeks, 5 days a week, and the running training time was fixed between 14:00–16:00. The state of the mice was regularly observed, and body weight changes were recorded during the running training. Considering the difference in running ability between young and old mice, we implemented special running training programs for mice of different ages. The treadmill adaptive training was performed as described above. Formal running training was started on the fourth day. For the next 8 weeks, the maximum running speed test was recorded each week before the weekly running exercise training, and 70% of the average maximum running speed of each group was used as their weekly running speed. The above exercise intensity exceeds the maximal exercise intensity at which blood lactate concentration remains constant [26, 94]. The maximum running speed test regime has been previously described [30].

### Morris water maze

Morris water maze (MWM) was performed to examine spatial learning and memory capacity. The test was performed in a grey circular pool (diameter 150 cm) divided into four equal quadrants, the pool is filled with water (26 °C) and has a depth of 16 cm, water was made opaque with non-toxic white paint to prevent animals from seeing the platform. A camera was mounted directly above the center of the pool, which was connected to a computerized recording system that included a tracking program (S-MART 3.0; PanLab Co., Barcelona, Spain), which was able to track and record mice swimming path. The test included four platform trials daily for 5 consecutive days (acquisition phase), followed by a probe trial test on the sixth day. During the acquisition phase, the mice were placed into a water pool in a quadrant with a non-placed platform and allowed to find the hidden platform for 60 s. If the mice did not find the platform within 60 s, it was gently guided to the platform for 15 s to help it remember the location of platform at end of each trial. This trial was repeated four times daily until no significant improvement in performance was identified. On day 6, the platform was removed and each mouse was allowed to swim freely for 60 s from the same position, opposite to the platform location. The time spent in target quadrant and swimming were recorded.

### Lactate measurements

Lactate levels in tissues were measured using the L-lactate assay kit (Cat# *A019-2–1,* Jiancheng, Nanjing, China) according to the manufacturer’s protocol. Lactate levels in murine tail vein blood were determined using a blood lactate analyzer (Vivachek, Hangzhou, China).

### HE staining and Nissl staining

To evaluate neuron damage, hematoxylin and eosin (HE) (Solarbio Science and Technology, Beijing, China) and Nissl staining (Solarbio Science and Technology, Beijing, China) were carried out according to the manufacturer’s instructions. The mounted slides were then examined and photographed using a OPLENIC professional camera system (Japan). The number of Nissl-positive neurons was counted using ImageJ software.

### Immunofluorescent and immunohistochemical

For immunofluorescent staining, specimens were deparaffinized and rehydrated before Immunofluorescent staining. Paraffin sections were blocked with goat blocking serum, incubated with primary antibodies (anti-Iba1, Abcam, Cambridge, UK,1:400 dilution; anti-Arg1, Servicebio, Wuhan, China 1:200 dilution; anti-Pan Kla, PTM BIO, Hangzhou, China, 1:200 dilution; anti-H3K18la, PTM BIO, Hangzhou, China, 1:200 dilution), incubated with secondary antibodies (Alexa Fluor 488 conjugated (green), Alexa Fluor 594 conjugated (red), ProteinTech, Wuhan, China), and then mounted on slides. The mounted slides were examined and photographed using an Olympus BX53 fluorescence microscope (Olympus Corporation). The mean fluorescence intensity was measured by Image J software, Iba1 positive and Arg1 positive cells were counted manually.

For immunohistochemical staining, specimens were deparaffinized and rehydrated before immunohistochemical staining, the subsequent experimental protocol was carried out according to the instructions of the DAB kit and the IHC kit (MXB Bio-company, Fuzhou, China). The mounted slides were then examined and photographed using a OPLENIC professional camera system (Japan). Microglia were identified by Iba1-positive immunoreactivity and Iba1-positive area was quantified using the ImageJ software.

### Oligomeric Aβ_1-42_

Oligomeric Aβ_1-42_ was prepared from recombinant Aβ_1-42_ (GL Biochem, Shanghai, China) peptide as previously described [95]. Briefly, the Aβ peptide was monomerized in 1 mM hexafluoroisopropanal (HFIP) (Aladdin, Shanghai, China) and the HFIP was evaporated using the Sterile fume hood. Oligomeric Aβ was prepared by resuspending the monomeric peptide in 5 mM dimethylsulfoxide (DMSO) (Solarbio Science and Technology, Beijing, China). The peptide was then diluted to 100 μM in phenol red-free F12 media (Gibco, CA, USA). The Aβ_1-42_ solution was incubated for 24 h on ice at 4 °C to allow oligomer formation.

### Cell culture

The BV2 microglia cell line was purchased from the Cell Bank of the Chinese Academy of Science (Shanghai, China). The BV2 cells were cultured in 1640 medium (RPMI-1640, Gibco, CA, USA) supplied with 10% fetal bovine serum (FBS, Clark, Richmond, VA, USA) at 37 °C with 5% CO_2_. BV2 cells were pretreated with sodium lactate solution (NaLA, 25 μM) (> 99%, Sigma-Aldrich, MO, USA) for 2 h, and then Aβ_1-42_ (25 μM, GL Biochem, Shanghai, China) was added for 2 h, 6 h, 12 h, and 24 h, or LPS (200 mg/ml, Sigma-Aldrich, MO, USA) was added for 24 h.

### Western blotting and immunocytochemistry

Western blotting was performed using standard methods. Collected cells were washed and lysed in RIPA lysis buffer containing protease and phosphatase inhibitor cocktails and phenyl-methylsulphonyl fluoride (PMSF) (Beyotime Biotechnology, Shanghai, China). The samples were loaded per lane and separated by 12.5% SDS-PAGE (Servicebio, Wuhan, China), and blocked with 5% non-fat blocking grade milk. Primary antibody (anti-Pan Kla, PTM BIO, Hangzhou, China, 1:2000 dilution; anti-α-Tubulin, ProteinTech, Wuhan, China, 1:1000 dilution, anti-p300, Affinity, Liyang, China, 1:2000 dilution) was added for overnight incubation, followed by TBST washing. ECL was used for illumination and development in a dark room after incubation of nitrocellulose membrane with secondary antibody (1:8000 dilution). Protein expression in Western blots was assessed and normalized by densitometry using ImageJ software.

Immunocytochemistry staining was performed using standard methods. The details were described elsewhere [96]. Briefly, the processed samples were incubated overnight with primary antibodies (anti-Pan Kla, PTM BIO, Hangzhou, China, 1:300 dilution). After washes in TBST, the samples were incubated with secondary species-specific antibodies (Alexa Fluor 594 conjugated, ProteinTech, Wuhan, China, 1:1000 dilution), cell nuclei were counterstained with Hoechst 33,342 (Beyotime Biotechnology, Shanghai, China).

### siRNA transfection

The siRNA or negative control were transfected using Lipo2000 (Introgen, USA) by the standard protocol. For siRNA transfection, cells were transfected at 80% confluency in 6-well plates with 75 nM control siRNA (con si) or p300-targeting siRNA (p300 si) (sense, 5’- CCAGAUGAAUUAAUCAACUTT-3’; antisense, 5’- AGUUGAUUAAUUCAUCUGGTT-3’) using Lipo2000 transfection reagents according to the manufacturer’s instructions. BV2 cells were seeded 14–18 h prior to transfection, and the cell monolayer reached the desired 70 to around 80% confluence at the time of transfection. 6 h after transfection, the cells were treated with normal training conditions (1640 medium with 10% FBS) or add sodium Aβ_1-42_(25 μM)/NaLA (25 μM) for 24 h.

### Real-time quantitative polymerase chain reaction (qPCR)

Collected cells were lysed with Trizol Reagent (Invitrogen, CA, USA). TransScript first-strand cDNA synthesis SuperMix (TransGen, Beijing, China) was used to make cDNA, qPCR with SYBR-Green mix (Roche, Basel, Switzerland) to measure the expression of genes. The following primers were used: β-actin, ACCTTCTACAATGAGCTGCG/CTGGATGGCTACGTACATGG; IL-1β, ACGGACCCCAAAAGATGAAG/TTCTCCACAGCCACAATGAG; Arg1, TCACCTGAGCTTTGATGTCG/TTCCCAAGAGTTGGGTTCAC; VEGF, CCACGACAGAAGGAGAGCAGAAGTCC/CGTTACAGCAGCCTGCACAGCG. P300, GAACAGGAAGAGGAAGAGAGGAAAC/TGAGAAAGGTCATTAGACACATTGG. The mRNA expression levels were normalized to β-actin.

### Statistical analysis

Analyses were conducted with GraphPad Prism 8.0.2 Software. Data were expressed as the mean ± SEM and were compared by using student’s t-test between two groups and one-way or two-way ANOVA with Tukey’s correction for multiple group comparison. A *p* value of < 0.05 was considered to be statistically significant.

### Supplementary Information

**Additional file 1:** **Supplementary Fig. 1. **Maximum running speed test in mice. The maximum running speed (m/min) of the mice in each group was detected weekly using a mouse treadmill within 8 weeks (*n *=8-12 per group). Data are means ± SEM.

**Additional file 2:** **Supplementary Fig. 2. **Lactate levels in the mice following running exercise or sodium lactate injection. (A) Lactate levels in the plasma of mice following exercise training (*n *=6 per group). (B) Lactate levels in the brain tissue homogenate of mice following exercise training (*n*=6 per group). (C) Timecourse of plasma lactate after subcutaneous injection (*n *=5 per group). (D) Lactate levels in the plasma of mice following sodium lactate injection (*n*=6 per group). Date are means ± SEM. *p < 0.05, **p < 0.01,***p < 0.001. Statistical analysis was performed using t tests.

**Additional file 3:** **Supplementary Fig. 3. **Running exercise induces Histone H3 lactylation in 4-month-old mice, 15-month-old mice and 20-month-old mice. (A) Double immunofluorescent staining of Iba1 (green) and Pan-Kla (red) of mice in in mice of different months of age (magnification 400×). (B) Representative images of H3K18la immunofluorescence staining in mice of different months of age (magnification 400×).

**Additional file 4:** **Supplementary Fig. 4. **Lactate regulates BV2 cell phenotype through lactylation. (A-B) Expression of Arg1 and VEGF at different time points in BV2 cells incubated with LPS and LPS+NaLA (*n* = 3 per group, normalized to expression at 0 hour). (C-D) Western blot shows the levels of Histone Kla in BV2 cells incubated with LPS or LPS+NaLA for 24 h. (*n* = 3 per group, normalized to the untreated sample). Date are means ± SEM. *p < 0.05, **p < 0.01. Statistical analysis was performed using unpaired Student’s two-tailed t-tests (A, B) or one-way ANOVA, followed by Tukey’s multiple comparisons test (D).

## Data Availability

Data will be made available on request.
