# Supplementary figures and images for: Exercise improves cognitive dysfunction and neuroinflammation in mice through Histone H3 lactylation in microglia
Source: Immun Ageing. 2023 Nov 17;20:63. doi: 10.1186/s12979-023-00390-4 (PMC10655345; doi:10.1186/s12979-023-00390-4)

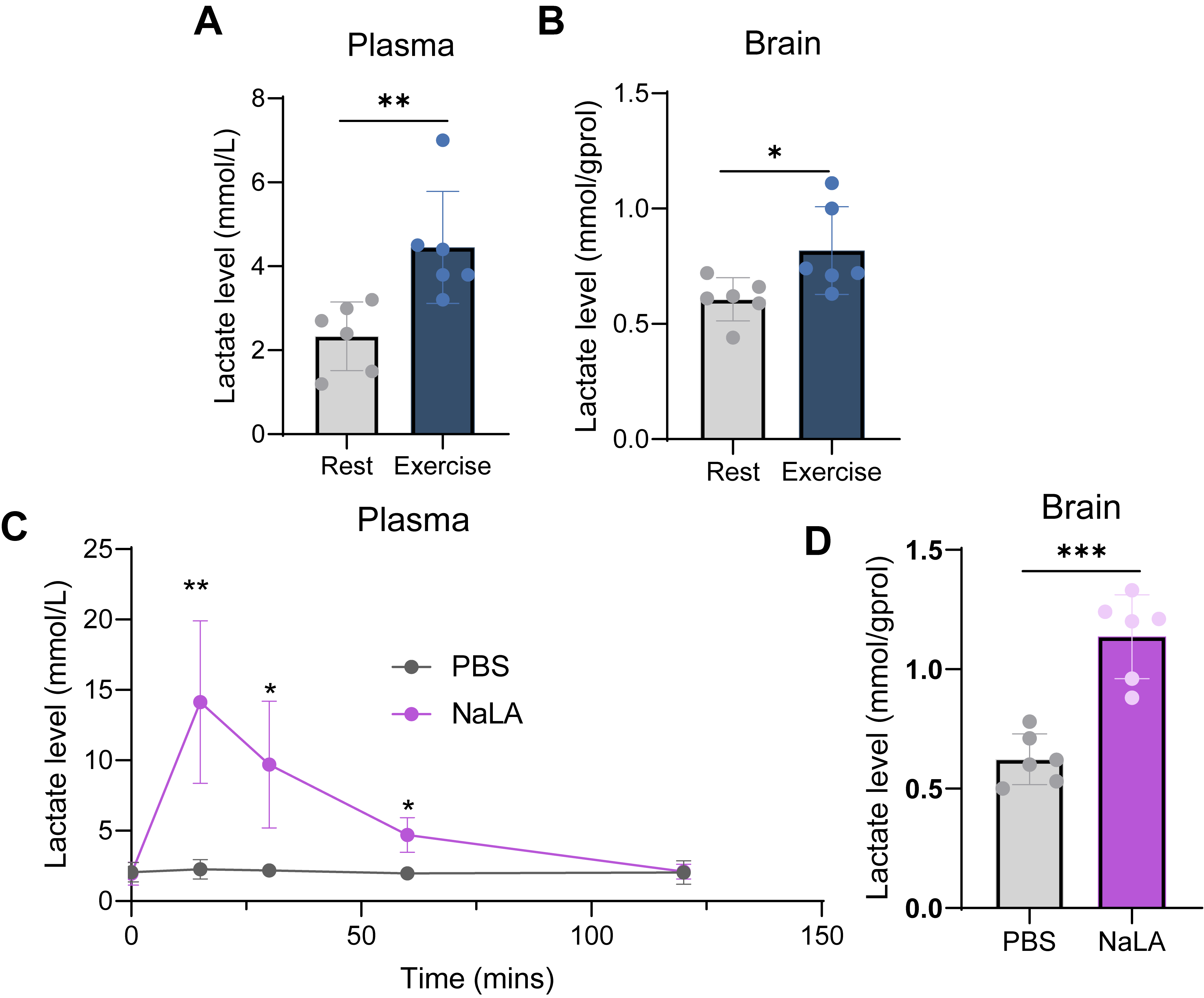

Supplement: Supplementary file 2 — Additional file 2: Supplementary Fig. 2. Lactate levels in the mice following running exercise or sodium lactate injection. (A) Lactate levels in the plasma of mice following exercise training (n =6 per group). (B) Lactate levels in the brain tissue homogenate of mice following exercise training (n=6 per group). (C) Timecourse of plasma lactate after subcutaneous injection (n =5 per group). (D) Lactate levels in the plasma of mice following sodium lactate injection (n=6 per group). Date are means ± SEM. *p < 0.05, **p < 0.01,***p < 0.001. Statistical analysis was performed using t tests. [file 12979_2023_390_MOESM2_ESM.tif]

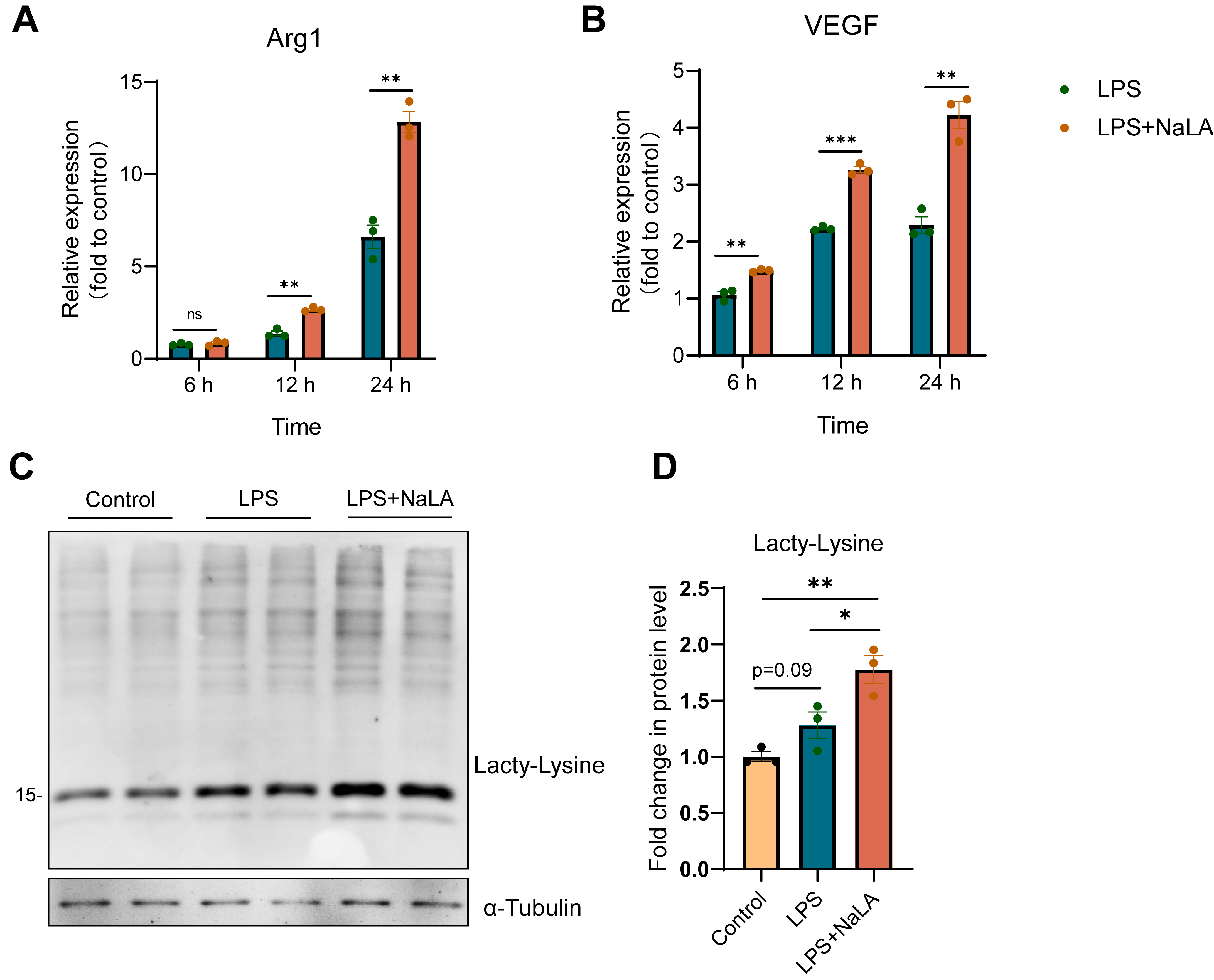

Supplement: Supplementary file 4 — Additional file 4: Supplementary Fig. 4. Lactate regulates BV2 cell phenotype through lactylation. (A-B) Expression of Arg1 and VEGF at different time points in BV2 cells incubated with LPS and LPS+NaLA (n = 3 per group, normalized to expression at 0 hour). (C-D) Western blot shows the levels of Histone Kla in BV2 cells incubated with LPS or LPS+NaLA for 24 h. (n = 3 per group, normalized to the untreated sample). Date are means ± SEM. *p < 0.05, **p < 0.01. Statistical analysis was performed using unpaired Student’s two-tailed t-tests (A, B) or one-way ANOVA, followed by Tukey’s multiple comparisons test (D). [file 12979_2023_390_MOESM4_ESM.tif]
